# Embitterment symptoms and comorbid psychopathology in an anxiety outpatient clinic: frequency, clinical distribution, and psychosocial correlates

**DOI:** 10.3389/fpsyt.2026.1874299

**Published:** 2026-07-24

**Authors:** Bahar Arslan, Süheyla Ünal

**Affiliations:** 1 Dept of Psychiatry, Republic of Türkiye (TC) Saglik Bakanligi Elazig Ruh Sagligi ve Hastaliklari Hastanesi, Elâzığ, Türkiye; 2 Dept of Psychiatry, Republic of Türkiye (TC) Saglik Bakanligi Sağlık Bilimleri Üniversitesi (SBU) Bursa Yuksek Ihtisas Egitim ve Arastirma Hastanesi, Bursa, Türkiye

**Keywords:** anxiety disorders, embitterment, hostility, posttraumatic embitterment disorder, psychiatric comorbidity, psychosocial stressors

## Abstract

**Objective:**

Posttraumatic embitterment disorder (PTED) has been proposed as a clinical construct characterized by persistent feelings of injustice, resentment, helpless anger, and intrusive embitterment-related cognitions following negative life experiences perceived as unjust, humiliating, or irreversible. The present study aimed to determine the frequency and clinical distribution of clinically relevant embitterment symptoms and PTED-compatible presentations.

**Methods:**

This cross-sectional study included 344 participants, consisting of 315 consecutive patients attending an anxiety outpatient clinic at a tertiary psychiatric center and 29 healthy control volunteers. Psychiatric diagnoses were established according to DSM-5 criteria through face-to-face clinical interviews, while PTED-related symptoms were evaluated using the clinical characteristics and standardized symptom criteria proposed by Linden and colleagues. Participants completed the Posttraumatic Embitterment Disorder Self-Rating Scale (PTED Scale), the Brief Symptom Inventory (BSI), the Buss–Perry Aggression Questionnaire (BPS), the Life Problems Screening List, and a structured sociodemographic assessment form.

**Results:**

On the PTED Self-Rating Scale, 178 participants (56.5%) obtained a mean score exceeding the established threshold of 2.5, indicating clinically significant embitterment. Of these, 114 participants (36.2% of the total sample) additionally met the chronicity criterion (symptom duration ≥6 months), consistent with a PTED-compatible profile as proposed by Linden et al. (2004). Embitterment severity was significantly associated with hopelessness, hostility, anger expression, somatization, negative self-perception, interpersonal relationship problems, occupational stress, financial stress, parenting stress, marital stress, childhood abuse, and dysfunctional family structure.

**Conclusion:**

The present findings suggest that PTED-compatible symptom patterns may represent a clinically meaningful and underrecognized cognitive-emotional syndrome in psychiatric outpatient settings. Further longitudinal and cross-cultural studies are needed to clarify the diagnostic boundaries, phenomenology, and therapeutic implications of PTED-compatible presentations.

## Introduction

Negative life events that are perceived as unjust, humiliating, irreversible, or uncontrollable may lead to a broad range of psychological responses. In some individuals, such experiences are followed by transient emotional distress, whereas in others they may contribute to persistent resentment, helpless anger, social withdrawal, hostility, and loss of trust. Within this spectrum, embitterment has received increasing clinical attention as a psychological response centered on perceived injustice, humiliation, and the inability to restore a previous sense of fairness or personal dignity (1, 2).

PTED-compatible presentations have been proposed to describe a specific clinical pattern that develops after negative life events that are not necessarily life-threatening but are experienced as deeply unfair, humiliating, or incompatible with the individual’s core values. A single negative life event perceived as unjust or irreversible may initiate persistent embitterment reactions in vulnerable individuals. Recollections of the event are often accompanied by emotional arousal, grief, anger, intrusive thoughts, and recurrent blaming of the triggering event for current unfavorable circumstances. Over time, beliefs concerning fairness and interpersonal trust may become increasingly disrupted, resulting in chronic resentment, helplessness, social withdrawal, hostile attitudes, revenge fantasies, self-blame, and diminished self-esteem (1, 3–5).

Although PTED is not yet recognized as a distinct diagnostic category in DSM-5-TR or ICD-11 (6, 7), embitterment-related presentations can overlap with existing trauma- and stressor-related diagnoses. When clinically appropriate, these presentations can be coded within existing frameworks: acute stress reaction (ICD-11: QE84/DSM: 308.3), adjustment disorder with mixed emotional and conduct disturbance (ICD-11: 6B43/DSM: 309.4), or other specified trauma- and stressor-related disorder (ICD-11: 6B4Y/DSM: 309.89). Therefore, PTED-compatible presentations should be viewed as a proposed clinical construct that describes a specific pattern of symptoms, rather than the sole way embitterment psychopathology can manifest. The key clinical question is not simply whether PTED is formally listed as a separate disorder, but how embitterment symptoms can be distinguished from other stress-related, adjustment-related, anxiety-related, and depressive presentations.

Unlike PTSD, which involves fear-related hyperarousal, avoidance, and threat processing, PTED-compatible presentations appear to be characterized by a reduced sense of agency, feelings of helpless anger, a blocked sense of restitution, and persistent perceptions of injustice (2, 3). In contrast to ordinary disappointment or short-term resentment, PTED-compatible presentations are marked by ongoing embitterment, intrusive memories of the triggering event, helpless anger, perceived injustice, emotional distress when reminded of the event, and significant social and occupational impairment persisting for more than six months (2, 3, 5).

Embitterment-related psychopathology may be better conceptualized as a spectrum of stress-related responses rather than a single categorical entity (8). At the milder end of this continuum, embitterment may emerge as a transient emotional reaction following experiences of injustice, humiliation, betrayal, or significant loss. In some individuals, these reactions may present within recognized diagnostic frameworks such as acute stress reactions with embitterment features (7), adjustment disorders characterized by mixed emotional and behavioral disturbances, or other specified trauma- and stressor-related conditions (6, 7). At the more persistent and severe end of the spectrum, prolonged and clinically significant embitterment reactions may develop into PTED-compatible presentations characterized by chronic emotional pain, persistent perceptions of injustice, intrusive recollections, helplessness, functional impairment, and prolonged symptom persistence (1, 9). This dimensional perspective is consistent with conceptual models proposing that embitterment manifestations differ not only in severity but also in duration, functional impact, and degree of psychopathological organization (6).

This spectrum perspective is also compatible with current diagnostic systems, where different embitterment manifestations may appear under several stress-related diagnostic categories rather than a single unified diagnosis.

Its manifestations range from brief emotional reactions after perceived injustice to persistent psychological states marked by functional difficulties and ongoing preoccupation. PTED-compatible presentations represent the most severe and persistent form of this spectrum (8). Prior research has shown considerable overlap between embitterment and other psychiatric conditions, including generalized anxiety disorder, social anxiety disorder, panic disorder, depression, persistent depressive disorder, adjustment disorder, and trauma-related conditions (1–3, 10, 11).

Despite these defining characteristics, symptoms of embitterment are often overlooked in psychiatric practice. Research on embitterment among psychiatric outpatients in Türkiye is currently limited. Given that experiences of injustice, humiliation, social obligations, family conflicts, marital stress, and concerns about social standing can be influenced by cultural context, it is important to investigate embitterment-related manifestations in diverse cultural settings.

Therefore, this study aimed to explore embitterment-related manifestations in individuals attending a specialized anxiety outpatient clinic in Türkiye. Specifically, we sought to determine the frequency and clinical distribution of clinically relevant embitterment symptoms using the PTED Self-Rating Scale threshold, describe the psychiatric and psychosocial factors associated with embitterment, examine aggression, hostility, hopelessness, trauma-related complaints, and overall symptom severity in relation to embitterment status, and compare findings between patients and healthy controls where applicable.

## Materials and methods

### Participants and recruitment

The study was approved by the Ethics Committee of İnönü University (approval number: 2019/5-6) and conducted in accordance with the Declaration of Helsinki. It was conducted at the Anxiety Disorders Outpatient Clinic of İnönü University Turgut Özal Medical Center, a tertiary referral psychiatry center serving patients from the Eastern Anatolia region of Türkiye.

The final study sample consisted of 344 participants. The clinical sample comprised 315 consecutive individuals who presented to the anxiety outpatient clinic between February and March 2019 and agreed to participate. The comparison group comprised 29 healthy control volunteers recruited from individuals applying for routine medical reports. All participants provided written informed consent before participation. Participants younger than 18 years of age, individuals with cognitive impairment, illiterate participants, and individuals unable to speak Turkish were excluded from the study. The original study records did not specify separate counts for refusals, invitation-stage exclusions, or incomplete-data exclusions; therefore, the analyses were conducted using the available complete dataset for the variables included in each analysis.

### Clinical assessment

All psychiatric evaluations were conducted face to face by a psychiatrist according to DSM-5 diagnostic criteria. Because PTED is not included as an official DSM-5 diagnostic category, DSM-5 diagnoses were used to classify formal psychiatric diagnostic status, whereas PTED-related symptoms were evaluated separately. The clinical interview assessed current psychiatric diagnoses, age at symptom onset, symptom duration, trauma-related complaints, anxiety and depressive symptoms, psychosocial stressors, embitterment-related experiences, and associated functional impairment. After the clinical interview, participants completed the self-report questionnaires individually under supervision of the research team. No imputation procedure was applied; analyses were based on available complete data for each variable.

### PTED assessment and group classification

PTED-compatible symptoms were evaluated using two complementary sources of information: the Posttraumatic Embitterment Disorder Self-Rating Scale and clinical assessment of PTED-related symptom characteristics proposed by Linden and colleagues (5). The clinical assessment focused on a negative life event experienced as unjust, humiliating, or irreversible; persistent embitterment; helpless anger; intrusive recollections; hostile cognitions; distrust toward others; social withdrawal; revenge-related ideation; and impairment in functioning.

**Figure 1:**
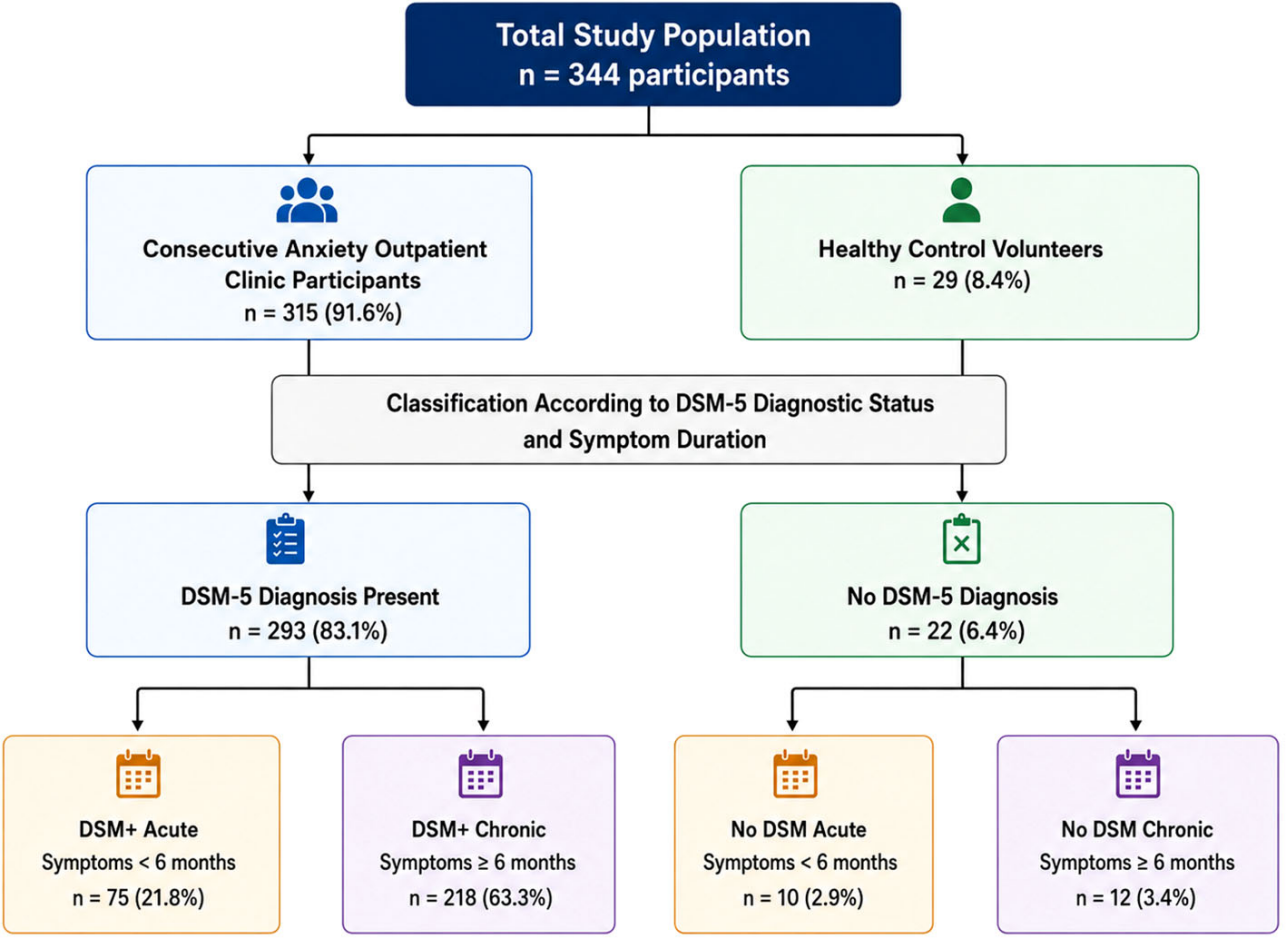
Participant flowchart showing recruitment, diagnostic classification, symptom chronicity, and subgroup allocation.

For scale-based classification, the PTED Self-Rating Scale was scored as a 19-item mean score, and the previously proposed cut-off of >2.5 was used to define clinically significant embitterment symptoms. A stricter PTED-compatible classification required the coexistence of a PTED mean item score >2.5 and symptom duration longer than six months. Participant recruitment and subgroup classification according to DSM-5 diagnostic status and symptom duration are illustrated in **Figure 1**.

### Measures

#### Sociodemographic data form

A sociodemographic data form developed by the researchers was used to obtain information on age, sex, marital status, education, occupation, place of residence, childhood caregiving characteristics, parenting style, family structure, breastfeeding history, separation from family during childhood, history of trauma or abuse, parental and personal medical history, age at symptom onset, symptom duration, and current psychosocial stressors.

#### Life problems screening list

The Life Problems Screening List was adapted from DSM-IV Axis IV psychosocial and environmental problem domains (12). It assessed life problems during the previous three months across seven domains: marital or opposite-sex relationship problems, occupational/school/home-work problems, financial problems, other interpersonal relationship problems, parenting-related problems, living-condition problems, and developmental or life-stage problems.

#### Posttraumatic embitterment disorder self-rating scale

The PTED Self-Rating Scale, developed by Linden and colleagues (9) and adapted into Turkish by Ünal and colleagues (13), consists of 19 self-report items rated on a 5-point Likert scale (0–4). Higher scores indicate greater embitterment severity. The scale assesses core embitterment and PTED-related phenomena such as perceived injustice, humiliation, intrusive recollections, helplessness, hopelessness, revenge-related thoughts, social withdrawal, avoidance, reduced functioning, and persistent painful memories. In the present analyses, the raw total score was divided by 19 to obtain the mean item score, and a cut-off of >2.5 was used to identify clinically significant embitterment symptoms.

#### Buss–Perry aggression questionnaire

The Buss–Perry Aggression Questionnaire (14) is a 29-item Likert-type self-report measure assessing aggression-related traits. It includes four subdimensions: physical aggression, verbal aggression, anger, and hostility. The Turkish validation study reported satisfactory reliability (Cronbach α = 0.85) (15). Total and subscale scores were treated as continuous indicators of aggression and hostility.

#### Brief symptom inventory

The Brief Symptom Inventory (BSI) (16, 17) is a 53-item self-report scale developed to assess general psychiatric symptom severity. The Turkish adaptation yielded a five-factor structure comprising anxiety, depression, somatization, negative self, and hostility (18). Total and subscale scores were analyzed as continuous measures.

No universally accepted pathological thresholds exist for BPS or BSI subscales; therefore scores were analyzed continuously, consistent with previous literature.

### Statistical analysis

Statistical analyses were performed using IBM SPSS Statistics version 23.0. Categorical variables were summarized as frequencies and percentages, and continuous variables as mean ± standard deviation. Pearson chi-square tests were used to compare categorical variables between groups. The Kolmogorov–Smirnov test was used to evaluate normality of continuous variables. Because several continuous variables were not normally distributed, Mann–Whitney U tests were used for pairwise group comparisons. Binary logistic regression analysis was performed to estimate adjusted associations with clinically significant embitterment; variables significant in bivariate analyses were entered into the regression model. Odds ratios were interpreted as exploratory adjusted associations rather than causal estimates, and the precision of estimates was evaluated using 95% confidence intervals. Statistical significance was set at p < 0.05.

## Results

### Sociodemographic characteristics

A total of 344 participants were included in the study, consisting of 315 anxiety outpatient participants and 29 healthy control volunteers. Among the anxiety outpatient participants, 197 (62.5%) were female and 118 (37.5%) were male. The mean age was 38.8 ± 15.2 years. Age, sex, education, residential location, and marital status were not significantly associated with clinically significant embitterment. Significant associations were found for excessive parental control, broken family structure, and childhood abuse. Psychosocial stressor analyses showed higher embitterment among participants reporting hopelessness, marital stress, occupational stress, financial stress, interpersonal relationship stress, and parenting stress (**Table 1**).

**Table 1:** Sociodemographic, family-related, and psychosocial characteristics associated with clinically significant embitterment.

Variable	Total n (%)/Mean ± SD	Embitterment n (%)/Mean ± SD	No Embitterment n (%)/Mean ± SD	p value
Sociodemographic variables				
Age (years)	38.8 ± 15.2	37.4 ± 14.7	40.6 ± 15.7	0.078
Female sex	197 (62.5)	111 (56.3)	86 (43.7)	0.940
Married	125 (39.7)	74 (59.2)	51 (40.8)	0.434
Higher education	184 (58.4)	111 (60.3)	73 (39.7)	0.268
Unemployed	110 (34.9)	64 (58.2)	46 (41.8)	0.974
Urban residence	262 (83.2)	148 (56.5)	114 (43.5)	0.988
Childhood and family variables				
Excessive parental control	80 (25.4)	57 (71.3)	23 (28.7)	0.006
Broken family structure	49 (15.6)	35 (71.4)	14 (28.6)	0.022
Childhood abuse	23 (7.3)	19 (82.6)	4 (17.4)	0.009
Childhood separation	39 (12.4)	25 (64.1)	14 (35.9)	0.307
Trauma history	156 (49.5)	91 (58.3)	65 (41.7)	0.517
Psychosocial stressors				
Hopelessness	218 (69.2)	148 (67.9)	70 (32.1)	<0.001
Marital stress	60 (19.0)	47 (78.9)	13 (21.1)	<0.001
Occupational stress	88 (27.9)	59 (66.7)	29 (33.3)	0.002
Financial stress	88 (27.9)	64 (72.7)	24 (27.3)	<0.001
Interpersonal relationship stress	86 (27.3)	63 (72.9)	23 (27.1)	<0.001
Parenting stress	55 (17.5)	41 (75.3)	14 (24.7)	<0.001
Changes in living conditions	58 (18.4)	37 (64.4)	21 (35.6)	0.072
Psychological breaking point	59 (18.7)	38 (64.8)	21 (35.2)	0.057

Continuous variables are presented as Mean ± SD; categorical variables as frequency (%). Chi-square or Mann–Whitney U tests were used where appropriate. Bold p values indicate statistical significance (p < 0.05).

### Embitterment frequency and diagnostic distribution

Clinically significant embitterment symptoms, defined by a PTED Self-Rating Scale mean item score >2.5, were observed in 178 of the 315 anxiety outpatient participants (56.5%). When the chronicity criterion of symptom duration longer than six months was added, 114 patients (36.2%) met the stricter PTED-compatible profile. Healthy controls were excluded from primary inferential analyses but retained for descriptive and exploratory subgroup comparisons.

**Figure 2:**
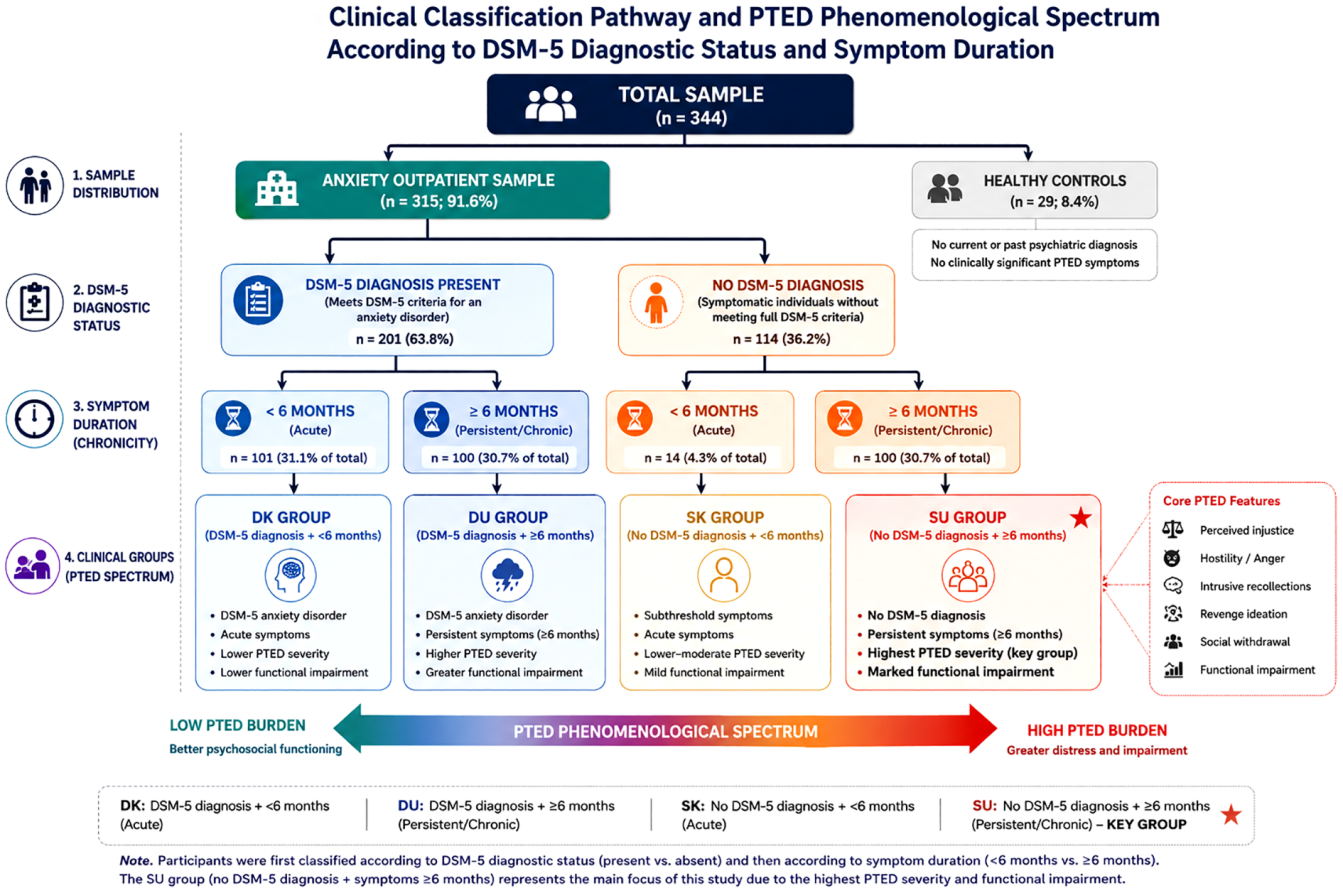
Distribution of clinically significant embitterment based on DSM-5 diagnostic status and symptom chronicity. A relatively high proportion of clinically significant embitterment was observed in individuals with symptoms lasting longer than six months who did not have a formal DSM-5 diagnosis (SU group), although this estimate should be interpreted cautiously because the subgroup was very small.

Among patients exceeding the PTED scale threshold, 162 of 178 (91.0%) had a DSM-5 diagnosis, whereas 16 of 178 (9.0%) were symptomatic patients without a formal DSM-5 diagnosis. According to subgroup classification, 75 patients (23.8%) had a DSM-5 diagnosis with acute symptom duration (DK), 218 (69.2%) had a DSM-5 diagnosis with chronic symptom duration (DU), 10 (3.2%) had no DSM-5 diagnosis with acute symptom duration (SK), and 12 (3.8%) had no DSM-5 diagnosis with chronic symptom duration (SU). Clinically significant embitterment symptoms were observed in 27/75 patients (36.0%) in the DK group, 135/218 (61.9%) in the DU group, 7/10 (70.0%) in the SK group, and 9/12 (75.0%) in the SU group (p < 0.001; **Figure 2**).

This exploratory finding may indicate a clinically relevant subgroup that is not fully captured by conventional diagnostic frameworks, but it should not be overinterpreted given the limited number of SU participants.

A relatively high proportional frequency was observed in symptomatic patients without a formal DSM-5 diagnosis whose symptoms persisted for over six months, indicating that clinically significant embitterment may manifest in patients whose distress does not align with standard diagnostic categories. Because this subgroup was small, the finding should be considered hypothesis-generating rather than definitive (**Table 2**).

**Table 2:** Distribution of embitterment among patients according to DSM-5 diagnosis.

DSM-5 diagnosis	Total n (%)	Embitterment n (%)	No embitterment n (%)	p
No formal DSM-5 diagnosis	22 (7.0)	16 (9.0)	6 (4.4)	0.004
Separation anxiety disorder	4 (1.3)	2 (1.1)	2 (1.5)	
Social anxiety disorder	5 (1.6)	2 (1.1)	3 (2.2)	
Panic disorder	30 (9.5)	13 (7.3)	17 (12.4)	
Generalized anxiety disorder	19 (6.0)	12 (6.7)	7 (5.1)	
Anxiety disorder due to another medical condition	36 (11.4)	18 (10.1)	18 (13.1)	
Other specified anxiety disorder	78 (24.8)	38 (21.3)	40 (29.2)	
Obsessive-compulsive disorder	25 (7.9)	16 (9.0)	9 (6.6)	
Trauma-related disorder	48 (15.2)	39 (21.9)	9 (6.6)	
Somatic symptom disorder	20 (6.3)	12 (6.7)	8 (5.8)	
Sleep, sexual behavior, and related disorders	28 (8.9)	10 (5.6)	18 (13.1)	

Chi-square analysis. p value refers to overall group comparison.

### Characteristic PTED-compatible symptom patterns

Item-level analysis demonstrated that embitterment-related psychopathology in the anxiety outpatient sample was characterized predominantly by persistent emotional suffering, intrusive recollections, helplessness, and impaired functioning. The most frequently endorsed severe manifestations were persistent emotional pain when recalling the event (61.9%), frequent painful recollections (61.0%), repetitive preoccupation with the triggering event (57.8%), and feeling deeply hurt or emotionally exhausted by the experience (56.8%). Severe helplessness and powerlessness were observed in more than half of participants (51.7%). Interestingly, revenge ideation represented one of the least frequently endorsed severe symptoms (27.3%), suggesting that embitterment in anxiety outpatients may be characterized less by overt retaliatory motivation and more by chronic emotional pain, helplessness, and intrusive recollections (**Table 3**).

**Table 3:** Distribution of severe endorsement across PTED-compatible symptom items in the anxiety outpatient sample (n = 315).

PTED symptom item	Severe endorsement n (%)	Mean score
Persistent emotional pain when recalling the event (P5)	195 (61.9)	3.66
Frequent painful recollections (P19)	192 (61.0)	3.60
Recurrent preoccupation with the event (P4)	182 (57.8)	3.53
Feeling deeply hurt and emotionally exhausted by the experience (P1)	179 (56.8)	3.48
Persistent unhappiness and irritability (P9)	172 (54.6)	3.41
Persistent negative impact on psychological well-being (P2)	163 (51.7)	3.36
Deterioration in physical health due to the event (P10)	163 (51.7)	3.34
Helplessness and powerlessness (P12)	163 (51.7)	3.37
Reduced motivation and will to live (P14)	159 (50.5)	3.24
Functional impairment in family/work roles (P17)	154 (48.9)	3.28
Increased irritability and anger (P15)	153 (48.6)	3.16
Perceived injustice/unfairness (P3)	148 (47.0)	3.11
Social withdrawal from activities and friends (P18)	148 (47.0)	3.19
Self-blame and self-directed anger (P7)	138 (43.8)	3.08
Wishing similar suffering upon responsible others (P13)	137 (43.5)	3.01
Persistent emotional distraction/inability to recover emotionally (P16)	130 (41.3)	3.00
Avoidance of reminders (P11)	129 (41.0)	2.86
Reduced motivation to make efforts (P8)	120 (38.1)	2.86
Revenge ideation (P6)	86 (27.3)	2.42

Severe endorsement was defined as scores of 4–5 on the 1–5 item response scale. These item-level results were derived from the dichotomized yes/no item table in the original thesis data; they should be interpreted as item endorsement frequencies rather than raw Likert severity scores.

Embitterment phenomenology in the anxiety outpatient sample appeared to be characterized predominantly by persistent emotional pain, intrusive recollections, helplessness, and functional impairment rather than revenge ideation alone.

### Psychopathology and symptom severity

All Buss–Perry Aggression Questionnaire and Brief Symptom Inventory total and subscale scores were significantly higher among participants with clinically significant embitterment compared with those without embitterment (all p < 0.001; **Table 4**). Participants with embitterment demonstrated elevated physical aggression, verbal aggression, anger, hostility, anxiety, depression, somatization, negative self-perception, and overall psychiatric symptom burden, indicating that embitterment was embedded in a broader psychopathological profile rather than representing an isolated emotional reaction.

**Table 4:** Buss–Perry aggression questionnaire and brief symptom inventory scores according to embitterment status.

Scale	n	Embitterment mean ± SD	No embitterment mean ± SD	p value
Buss–Perry aggression questionnaire				
Physical Aggression	315	20.6 ± 9.3	15.9 ± 6.7	<0.001
Verbal Aggression	315	13.6 ± 5.4	11.3 ± 4.4	<0.001
Anger	315	20.6 ± 5.9	17.2 ± 6.2	<0.001
Hostility	315	23.2 ± 8.0	16.9 ± 6.5	<0.001
BPS Total	315	78.0 ± 23.2	61.4 ± 19.3	<0.001
Brief symptom inventory				
Anxiety	315	21.6 ± 11.3	10.4 ± 8.5	<0.001
Depression	315	29.6 ± 11.8	14.6 ± 11.2	<0.001
Negative Self	315	22.2 ± 12.6	9.4 ± 8.8	<0.001
Somatization	315	16.0 ± 9.3	8.5 ± 6.2	<0.001
Hostility (BSI)	315	13.7 ± 6.5	6.7 ± 5.3	<0.001
BSI Total	315	105.8 ± 45.3	50.9 ± 35.3	<0.001

BPS, Buss–Perry Scale; BSI, Brief Symptom Inventory. Group comparisons were performed using Mann–Whitney U tests.

### Distribution of characteristic PTED-compatible symptoms across diagnostic subgroups

Item-level analyses demonstrated that participants with chronic symptomatic presentations, especially those in the DU group and, more tentatively, the smaller SU subgroup, showed higher frequencies of characteristic PTED-compatible symptoms compared to healthy controls. Persistent feelings of injustice, intrusive recollections, social withdrawal, hopelessness, distrust toward others, hostile cognitions, anger-related emotional preoccupation, and functional impairment were significantly more frequent in symptomatic groups (**Table 5**).

**Table 5:** Distribution of characteristic PTED-compatible symptoms across diagnostic and symptomatic subgroups.

PTED symptom item	DK %	DU %	SK %	SU %	Control %	p value
Persistent feelings of injustice/hurt	44.0	63.8	60.0	50.0	24.1	<0.001
Emotional arousal when reminded of the event	33.3	62.4	50.0	66.7	13.8	<0.001
Recurrent intrusive recollections	41.3	54.6	40.0	75.0	24.1	0.004
Social withdrawal and interpersonal distancing	52.0	66.1	70.0	66.7	31.0	0.003
Hopelessness and pessimistic future perception	53.3	63.3	60.0	66.7	34.5	0.038
Revenge-related thoughts and hostile cognitions	24.0	26.1	40.0	66.7	6.9	0.002
Distrust toward others	38.7	49.1	50.0	66.7	6.9	<0.001
Anger-related emotional preoccupation	34.7	48.6	50.0	66.7	13.8	0.001
Functional impairment associated with embitterment	44.0	59.2	60.0	75.0	17.2	<0.001

DK, DSM-5 diagnosis, acute symptom duration; DU, DSM-5 diagnosis, chronic symptom duration; SK, no DSM-5 diagnosis, acute symptom duration; SU, no DSM-5 diagnosis, chronic symptom duration. Chi-square analyses.

Clinically, these symptom patterns suggested that embitterment was associated not only with emotional distress but also with interpersonal and cognitive dysfunction. Prominent clinical characteristics included persistent feelings of injustice and humiliation, hopelessness, intrusive recollections related to negative life events, hostile attitudes, anger-related symptoms, interpersonal withdrawal, loss of trust toward others, and difficulties in emotional regulation. Many participants described recurrent preoccupation with stressful life events accompanied by chronic resentment and emotional arousal when reminded of the triggering experiences. These findings are consistent with the conceptualization of PTED-compatible manifestations as a recognizable cognitive-emotional pattern within anxiety-related distress, while also requiring replication in larger and diagnostically diverse samples.

### Logistic regression analysis

Binary logistic regression analysis was used to estimate factors independently associated with clinically significant embitterment after accounting for the variables entered into the model. Childhood abuse was associated with a higher likelihood of clinically significant embitterment (OR = 5.668, 95% CI = 1.079–29.777, p = 0.040), as was marital stress (OR = 3.684, 95% CI = 1.700–7.987, p = 0.001). These findings suggest that interpersonal adversity and current relational stress may contribute to vulnerability for embitterment-related symptoms. However, the wide confidence interval for childhood abuse indicates imprecision, most likely reflecting the small number of participants reporting childhood abuse; therefore, the magnitude of this association should be interpreted cautiously. Other variables examined in the final regression model did not show statistically significant independent associations (**Table 6**).

**Table 6:** Binary logistic regression analysis of factors associated with clinically significant embitterment.

Variable	β	SE	p	OR	95% CI
Family structure	−0.122	0.462	0.792	0.885	0.358–2.187
**Childhood abuse**	**1.735**	**0.846**	**0.040**	**5.668**	**1.079–29.777**
Hopelessness	0.106	0.376	0.778	1.112	0.532–2.321
**Marital stress**	**1.304**	**0.395**	**0.001**	**3.684**	**1.700–7.987**
Occupational stress	0.403	0.325	0.214	1.497	0.792–2.829
Financial stress	0.595	0.334	0.074	1.814	0.943–3.488
Interpersonal relations	0.509	0.333	0.126	1.664	0.867–3.194
BPS Physical Aggression	−0.010	0.042	0.813	0.990	0.912–1.075
BPS Verbal Aggression	−0.005	0.054	0.925	0.995	0.896–1.105
BPS Anger	−0.022	0.050	0.655	0.978	0.886–1.079
BPS Hostility	−0.005	0.054	0.925	0.995	0.896–1.105
BPS Total	0.009	0.030	0.767	1.009	0.952–1.069
Anxiety (BSI)	−0.193	0.155	0.211	0.824	0.608–1.116
Depression (BSI)	−0.120	0.156	0.440	0.887	0.653–1.203
Negative Self (BSI)	−0.129	0.142	0.365	0.879	0.666–1.161
Somatization (BSI)	−0.158	0.147	0.282	0.854	0.640–1.139
Hostility (BSI)	−0.084	0.152	0.580	0.919	0.682–1.238
BSI Total	0.169	0.145	0.244	1.184	0.891–1.573

OR, odds ratio; SE, standard error; CI, confidence interval. Dependent variable: presence of clinically significant embitterment (PTED Self-Rating Scale mean item score >2.5). Wide confidence intervals indicate lower precision and should be interpreted cautiously. Bold p values indicate statistical significance (p < 0.05).

## Discussion

The present study investigated embitterment-related manifestations in a tertiary anxiety outpatient population by distinguishing between clinically significant embitterment symptoms and PTED-compatible presentations. Several findings emerged. First, clinically significant embitterment symptoms were frequently observed among anxiety outpatients (56.5%), whereas substantially fewer participants (36.2%) fulfilled stricter PTED-compatible criteria requiring chronic symptom persistence. Second, embitterment-related psychopathology was characterized not only by elevated psychiatric symptom burden but also by a distinct phenomenological profile involving persistent emotional pain, intrusive recollections, helplessness, functional impairment, and perceived injustice. Third, clinically relevant embitterment symptoms were also observed among individuals who did not fully fit conventional diagnostic categories, although this subgroup finding should be considered exploratory because of small subgroup size.

These findings support the view that embitterment manifestations exist on a spectrum ranging from transient emotional reactions to chronic PTED-compatible presentations, consistent with previous conceptualizations of embitterment as a heterogeneous clinical construct rather than a single disorder entity.

Using the PTED Self-Rating Scale with a mean item cut-off of greater than 2.5, 56.5% of participants in the anxiety outpatient group exhibited clinically significant embitterment symptoms. This frequency is substantially higher than estimates reported for the general population (approximately 2–3%), which may be understandable given the characteristics of psychiatric outpatient samples (1, 2, 19). Kim et al. (20) reported clinically significant reactive embitterment symptoms in 37.8% of psychiatric patients, with no healthy participants meeting criteria. Heightened threat sensitivity, repetitive negative thinking, intolerance of uncertainty, and maladaptive appraisal styles commonly observed in anxiety disorders may increase vulnerability to persistent embitterment reactions following perceived injustice (1, 2, 19).

Cultural context may also contribute to the high frequency observed in this clinical sample. Previous findings from psychiatric outpatients in Türkiye suggest that culturally shaped relational self-construal may influence the way psychological distress is experienced and expressed (21). Although this evidence does not directly address embitterment, it supports the interpretation that cultural and relational patterns may shape the phenomenological expression and distribution of distress in Turkish psychiatric populations.

Linden and Rotter (8) described embitterment as a wide clinical spectrum; in their psychosomatic sample, 86.5% reported feelings of embitterment, suggesting that embitterment-related experiences may be relatively common in clinical populations when assessed directly. This spectrum perspective is consistent with our finding that embitterment symptoms were frequent in an anxiety outpatient setting and were associated with elevated hostility, depression, anxiety, somatization, negative self-perception, and overall psychiatric symptom severity.

Among participants experiencing embitterment, trauma-related disorders (including adjustment disorder) were the most common diagnoses, followed by other specified anxiety disorder and anxiety disorder due to another medical condition. These findings suggest that embitterment-related psychopathology may complicate existing diagnostic presentations rather than appearing as a completely isolated clinical entity, consistent with Linden et al. (2).

The frequency of embitterment among symptomatic individuals without formal DSM-5 diagnoses may be clinically important, but this observation should be interpreted as exploratory because the relevant subgroup was small. Acute embitterment reactions could be coded as acute stress reaction (ICD-11: QE84; DSM-5: 308.3), whereas more persistent presentations might align with adjustment disorder (ICD-11: 6B43) or other specified trauma- and stressor-related disorder (ICD-11: 6B4Y; DSM-5: 309.89). However, none of these categories fully capture the distinct characteristics of PTED-compatible presentations, which are specifically defined by persistent feelings of injustice, helpless anger, intrusive memories of the triggering event, thoughts of revenge, distrust of others, and social withdrawal lasting longer than six months (5). PTED-compatible presentations may represent a distinct phenomenological pattern within the broader spectrum of embitterment manifestations that is not fully captured by existing categorical systems (2, 22).

From a transdiagnostic perspective, the symptom distribution suggests that embitterment-related psychopathology in anxiety outpatients may be organized primarily around persistent emotional pain and agency-related dysfunction rather than revenge motivation alone. Severe emotional suffering, intrusive recollections, helplessness, and functional impairment were more frequently endorsed than overt revenge ideation, indicating that chronic emotional burden and impaired agency may constitute more central dimensions of embitterment-related presentations (23, 24).

The association between embitterment and hopelessness observed in the present study deserves particular attention. Linden and colleagues emphasized hopelessness, helplessness, irreversibility, and inability to receive help among the core phenomenological characteristics of the PTED construct (8). Kim et al. (20) demonstrated that embitterment significantly mediated the relationship between depression and suicidality, suggesting that embitterment may amplify emotional suffering through mechanisms involving chronic resentment, helplessness, and repetitive negative cognitive processing.

Childhood abuse was associated with a more than fivefold increase in the likelihood of embitterment symptoms in the present sample after adjustment for the variables entered into the regression model. Previous studies have similarly emphasized that interpersonal and human-induced traumatic experiences may generate intense helplessness, humiliation, anger, and embitterment because they undermine trust, empathy, and the expectation of being understood by others (25, 26). Within the PTED-compatible framework, the subjective meaning of the event and its violation of core assumptions may be more clinically relevant than the objective severity of the stressor itself (22, 27). However, because childhood adversity was assessed retrospectively and the confidence interval around this estimate was wide, recall bias, retrospective reconstruction processes, mood-congruent memory effects, and limited precision cannot be excluded. These findings should therefore be interpreted as adjusted associations requiring prospective investigation rather than evidence of causal relationships.

Additionally, socially mediated stressors such as humiliation, betrayal, chronic injustice, breach of trust, occupational stress, financial strain, interpersonal conflicts, and parenting-related stress may progressively diminish the individual’s sense of agency and ability to influence outcomes, thereby reinforcing helplessness, rumination, and chronic resentment (28, 29). Consistent with this interpretation, marital stress emerged as one of the strongest correlates of embitterment in the present study. Interpersonal trust, insecure attachment, and relational stress have previously been associated with vulnerability to embitterment reactions (30, 31).

From a clinical perspective, these findings suggest that assessment of embitterment-related symptoms may be particularly relevant among patients presenting with chronic interpersonal difficulties, persistent resentment, hopelessness, hostility, social withdrawal, treatment resistance, and symptom patterns that do not fit clearly within conventional DSM-5 categories. Importantly, embitterment-related psychopathology may contribute to apparent treatment resistance in routine psychiatric practice. Persistent resentment, rigid injustice-related cognitions, chronic rumination, interpersonal distrust, and hopelessness may weaken therapeutic alliance, impair emotional processing, and reduce engagement with conventional psychotherapeutic or pharmacological interventions. Consequently, unrecognized embitterment symptoms may partly explain why some patients continue to experience chronic distress despite receiving standard psychiatric treatment. Since embitterment has been associated with functional impairment, emotional exhaustion, and deterioration of interpersonal functioning, early recognition may facilitate more individualized therapeutic interventions and help clinicians identify clinically meaningful symptom patterns that might otherwise remain hidden within anxiety, depression, adjustment, or trauma-related diagnoses.

### Strengths of the study

The present study has several important strengths. It represents one of the early clinical investigations conducted in Türkiye examining the frequency, clinical distribution, clinical presentation, and psychiatric correlates of PTED-compatible presentations within a real-world psychiatric outpatient sample. The simultaneous evaluation of DSM-5 diagnoses, psychosocial stressors, childhood traumatic experiences, and symptom severity within the same clinical sample is a further strength. The inclusion of both DSM-5-diagnosed patients and symptomatic individuals without formal diagnoses enabled the identification of clinically significant embitterment symptoms that might otherwise remain underrecognized. The examination of embitterment within a specific sociocultural context where interpersonal obligations, family relationships, marital roles, and perceived injustice may shape symptom expression differently from previously studied populations adds cross-cultural value.

### Limitations of the study

Several limitations should be considered when interpreting these findings. First, the cross-sectional design precludes causal conclusions regarding the relationships among embitterment, psychiatric conditions, psychosocial stressors, and traumatic experiences. Second, the study was conducted in a single tertiary anxiety outpatient clinic, which may limit generalizability to broader psychiatric and community populations. Third, subgroup sizes were uneven, particularly among symptomatic participants without formal DSM-5 diagnoses; therefore, findings related to these smaller subgroups should be interpreted cautiously because of limited statistical power. Fourth, some regression estimates, especially the association involving childhood abuse, had wide confidence intervals, indicating limited precision and the need for replication in larger samples. Fifth, because PTED is not included as an official DSM-5 diagnostic category, embitterment-related phenomena were assessed using standardized PTED-compatible symptom descriptions and clinical characteristics proposed in the literature rather than structured diagnostic interviews. Sixth, childhood adversity was assessed retrospectively, making recall bias, retrospective reconstruction processes, and mood-congruent memory effects possible. Finally, the study did not directly assess cultural orientation or self-construal variables; therefore, interpretations regarding sociocultural influences on embitterment should be considered exploratory.

## Conclusion

This study highlights the frequency and clinical distribution of embitterment symptoms among anxiety outpatients in Türkiye, emphasizing that these represent a spectrum of embitterment manifestations—including persistent resentment, perceived injustice, interpersonal distrust, hopelessness, hostility-related symptoms, social withdrawal, and broader psychological distress—rather than a uniformly high PTED rate. A clinically important subgroup met the chronicity criterion consistent with a PTED-compatible presentation, although subgroup estimates should be interpreted cautiously because of small cell sizes. The frequency of clinically significant embitterment among symptomatic individuals without formal DSM-5 diagnoses suggests that PTED-compatible symptom patterns may remain underrecognized in routine psychiatric practice. Considering the negative impact of embitterment on treatment response, interpersonal functioning, and quality of life, clinicians may benefit from carefully evaluating PTED-compatible symptoms in psychiatric patients, particularly among individuals exposed to chronic interpersonal stressors and human-induced traumatic experiences. Future research should replicate findings in diverse samples and use longitudinal designs to clarify the phenomenology, diagnostic boundaries, and therapeutic implications of PTED-compatible presentations.

## Data Availability

The original contributions presented in the study are included in the article/supplementary material. Further inquiries can be directed to the corresponding author.
