# Supplementary material for: Embitterment symptoms and comorbid psychopathology in an anxiety outpatient clinic: frequency, clinical distribution, and psychosocial correlates
Source: Front Psychiatry. 2026 Jul 24;17:1874299. doi: 10.3389/fpsyt.2026.1874299 (PMC13447416; doi:10.3389/fpsyt.2026.1874299)
Supplement: Supplementary file 1 [file Table1.docx]

## **RESULTS**

### **Sample Characteristics**

The sample consisted of 344 individuals who applied to the anxiety outpatient clinic and was divided into five groups. The grouping was as follows: 21.8% (75 individuals) had a DSM-5 diagnosis and reported symptoms lasting less than 6 months; 63.3% (218 individuals) had a DSM-5 diagnosis and reported symptoms lasting longer than 6 months; 2.9% (10 individuals) had no DSM-5 diagnosis but reported symptoms lasting less than 6 months; 3.4% (12 individuals) had no DSM-5 diagnosis but reported symptoms lasting longer than 6 months; and 8.4% (29 individuals) consisted of healthy control subjects. The sociodemographic characteristics of the groups are presented in Table 4.1.

***Tablo 4.1. Sosyodemografik özelliklerin gruplar arasında karşılaştırılması***

| Variables | DK % (n) | DU % (n) | SK % (n) | SU % (n) | K % (n) | p |
| --- | --- | --- | --- | --- | --- | --- |
| Age (M ± SD) | 41.5 ± 16 | 38.1 ± 12.3 | 35 ± 4.4 | 33 ± 13.7 | 33.5 ± 12.8 | .458 |
| Gender |  |  |  |  |  | .254 |
| Female | 60.0 (45) | 65.1 (142) | 60.0 (6) | 33.3 (4) | 65.5 (19) |  |
| Male | 40.0 (30) | 34.9 (76) | 40.0 (4) | 66.7 (8) | 34.5 (10) |  |
| Marital status |  |  |  |  |  | .622 |
| Married | 45.3 (34) | 37.6 (82) | 30.0 (3) | 50.0 (6) | 34.5 (10) |  |
| Single | 54.7 (41) | 62.4 (136) | 70.0 (7) | 50.0 (6) | 65.5 (19) |  |
| Education level |  |  |  |  |  | .553 |
| Illiterate | 2.7 (2) | 5.5 (12) | 0.0 (0) | 0.0 (0) | 3.4 (1) |  |
| Primary school | 29.3 (22) | 38.5 (84) | 50.0 (5) | 50.0 (6) | 20.7 (6) |  |
| High school | 28.0 (21) | 27.1 (59) | 20.0 (2) | 25.0 (3) | 31.0 (9) |  |
| University | 40.0 (30) | 28.9 (63) | 30.0 (3) | 25.0 (3) | 44.8 (13) |  |
| Occupation |  |  |  |  |  | .030* |
| Unemployed | 12.0 (9) | 8.7 (19) | 10.0 (1) | 33.3 (4) | 13.8 (4) |  |
| Paid employee | 14.7 (11) | 14.2 (31) | 20.0 (2) | 0.0 (0) | 31.0 (9) |  |
| Self-employed/irregular | 17.3 (13) | 23.9 (52) | 40.0 (4) | 50.0 (6) | 17.2 (5) |  |
| Housewife | 24.0 (18) | 30.3 (66) | 20.0 (2) | 0.0 (0) | 24.1 (7) |  |
| Retired | 8.0 (6) | 10.6 (23) | 0.0 (0) | 8.3 (1) | 3.4 (1) |  |
| Student | 24.0 (18) | 12.4 (27) | 10.0 (1) | 8.3 (1) | 10.3 (3) |  |
| Age of onset |  |  |  |  |  | .<001** |
| Childhood | 10.7 (8) | 15.6 (34) | 0.0 (0) | 66.7 (8) | 0.0 (0) |  |
| Young adulthood | 29.3 (22) | 23.4 (51) | 0.0 (0) | 16.7 (2) | 0.0 (0) |  |
| Adulthood | 36.0 (27) | 40.8 (89) | 100.0 (10) | 16.7 (2) | 0.0 (0) |  |
| Older age | 24.3 (18) | 20.2 (44) | 0.0 (0) | 0.0 (0) | 0.0 (0) |  |
| Duration of illness |  |  |  |  |  | .<001** |
| 0–1 month | 29.3 (22) | 0.0 (0) | 100.0 (10) | 0.0 (0) | 0.0 (0) |  |
| 1–6 months | 70.7 (53) | 0.0 (0) | 0.0 (0) | 0.0 (0) | 0.0 (0) |  |
| 6 months–1 year | 0.0 (0) | 23.9 (52) | 0.0 (0) | 8.3 (1) | 0.0 (0) |  |
| 1–2 years | 0.0 (0) | 17.4 (38) | 0.0 (0) | 8.3 (1) | 0.0 (0) |  |
| 2–5 years | 0.0 (0) | 19.3 (42) | 0.0 (0) | 25.0 (3) | 0.0 (0) |  |
| 5–10 years | 0.0 (0) | 10.1 (22) | 0.0 (0) | 8.3 (1) | 0.0 (0) |  |
| ≥10 years | 0.0 (0) | 29.4 (64) | 0.0 (0) | 50.0 (6) | 0.0 (0) |  |
| Family structure |  |  |  |  |  | .001* |
| Broken | 13.3 (10) | 14.7 (32) | 10.0 (1) | 50.0 (6) | 0.0 (0) |  |
| Intact | 86.7 (65) | 85.3 (186) | 90.0 (9) | 50.0 (6) | 100.0 (29) |  |
| Mother’s employment |  |  |  |  |  | .003* |
| Not working | 93.3 (70) | 81.7 (178) | 90.0 (9) | 50.0 (6) | 86.2 (25) |  |
| Working | 6.7 (5) | 18.3 (40) | 10.0 (1) | 50.0 (6) | 13.8 (4) |  |
| Personal psychiatric history |  |  |  |  |  | .033* |
| No | 89.3 (67) | 82.6 (180) | 60.0 (6) | 66.7 (8) | 93.1 (27) |  |
| Yes | 10.7 (8) | 17.4 (38) | 40.0 (4) | 33.3 (4) | 6.9 (2) |  |
| History of abuse |  |  |  |  |  | .252 |
| No | 94.7 (71) | 92.2 (201) | 100.0 (10) | 83.3 (10) | 100.0 (29) |  |
| Yes | 5.3 (4) | 7.8 (17) | 0.0 (0) | 16.7 (2) | 0.0 (0) |  |

Note. Values are presented as percentage (n). M = mean; SD = standard deviation. * p < .05, ** p < .001.

### **Table 4.1. Comparison of Sociodemographic Characteristics Between Groups** Chi-square test p<0.05 DK: DSM-5 diagnosis with disease duration less than 6 months DU: DSM-5 diagnosis with disease duration longer than 6 months SK: No DSM-5 diagnosis with symptoms less than 6 months SU: No DSM-5 diagnosis with symptoms longer than 6 months K: Control

In the symptomatic group, where higher embitterment rates were expected, and in the DU group (DSM-5 diagnosed with symptoms longer than six months), the highest occupational percentage was housewives at 30.3% (66 individuals). In the symptomatic groups with short- and long-term symptoms, private sector employees constituted 40% (4 individuals) and 50% (6 individuals), respectively. There was a difference between occupational groups according to the chi-square test; however, since the proportion of cells with expected counts less than five was 63.5%, the result was not considered reliable. Apart from this, no significant differences were found between groups in other sociodemographic variables.

The mean age of the sample was 38.8 ± 15 years, ranging from a minimum of 18 to a maximum of 86 years. A significant difference was found between groups in terms of age of onset (p=0.000). In the DU group, the highest onset was during adulthood at 40.8% (89 individuals), whereas in the SU group, which is another important group in terms of embitterment, 66.7% (8 individuals) had onset during childhood. Disease duration also differed significantly between groups (p=0.000). Particularly, disease duration longer than 10 years was 29.4% (64 individuals) in the DU group and 50% (6 individuals) in the SU group.

Among those with a fragmented family structure, the highest proportion was observed in the SU group at 50% (6 individuals), whereas the lowest proportion was 0% in the control group. Family structure differed significantly between groups (p=0.001). Maternal employment status differed significantly between groups, with rates of 18.3% (40 individuals) in the DU group and 50% (6 individuals) in the SU group (p=0.003). The number of individuals reporting the presence of a serious personal illness was highest in the DU group at 17.4% (38 individuals), and this difference was statistically significant (p=0.033). No significant differences were found between groups in terms of separation from family and abuse.

### **Table 4.2. Comparison of Trauma Experience and Presence of PTED Between Groups**

| Group | DK % (n) | DU % (n) | SK % (n) | SU % (n) | K % (n) | p |
| --- | --- | --- | --- | --- | --- | --- |
| TSKB |  |  |  |  |  | .<001** |
| No | 64.0 (48) | 38.1 (83) | 30.0 (3) | 25.0 (3) | 89.7 (26) |  |
| Yes | 36.0 (27) | 61.9 (135) | 70.0 (7) | 75.0 (9) | 10.3 (3) |  |

Note. Values are presented as percentage (n). Chi-square test was used. ** p < .001.

Chi-square test p<0.05

In the DK group (DSM-5 diagnosis with symptoms less than 6 months), embitterment was observed in 7.8% (27 individuals), and in the SK group (symptomatic without DSM-5 diagnosis, less than 6 months), it was observed in 2% (7 individuals). In the SU and DU groups (those scoring significantly on the embitterment scale and reporting symptoms longer than 6 months, thus meeting disorder criteria), embitterment disorder was observed in 41.8% (144 individuals). In the DU group (DSM-5 diagnosis with embitterment disorder), the rate was 39.2% (135 individuals). A significant difference was found between groups in terms of embitterment (p=0.000).

DSM-5 anxiety subgroups were examined as separation anxiety, social phobia, panic disorder, generalized anxiety disorder, anxiety due to a medical condition, other specified anxiety disorder, obsessive-compulsive disorder, trauma-related disorders, somatic symptom disorder, and other DSM-5 diagnoses. Although a significant difference was found between groups in terms of DSM-5 diagnoses, the results were not considered reliable because more than 30% of the cells had expected counts less than five. In the DK group, the most frequent diagnosis was other specified anxiety disorder at 54.7% (41 individuals). In the DU group, trauma-related disorders were the most common diagnosis at 20.2% (44 individuals). In this group, which is important in terms of embitterment disorder, the second most frequent diagnosis was other specified anxiety disorder at 17% (37 individuals), followed by anxiety due to a medical condition at 14.2% (31 individuals).

### **Table 4.3. Comparison of Life Stressors Between Groups**

| Variables | DK % (n) | DU % (n) | SK % (n) | SU % (n) | K % (n) | p |
| --- | --- | --- | --- | --- | --- | --- |
| Marital problems |  |  |  |  |  | .775 |
| No | 78.7 (59) | 74.3 (162) | 80.0 (8) | 83.3 (10) | 82.8 (24) |  |
| Yes | 21.3 (16) | 25.7 (56) | 20.0 (2) | 16.7 (2) | 17.2 (5) |  |
| Work-related problems |  |  |  |  |  | .135 |
| No | 52.0 (39) | 60.6 (132) | 60.0 (6) | 50.0 (6) | 79.3 (23) |  |
| Yes | 48.0 (36) | 39.4 (86) | 40.0 (4) | 50.0 (6) | 20.7 (6) |  |
| Financial problems |  |  |  |  |  | .045* |
| No | 73.3 (55) | 58.3 (127) | 60.0 (6) | 50.0 (6) | 79.3 (23) |  |
| Yes | 26.7 (20) | 41.7 (91) | 40.0 (4) | 50.0 (6) | 20.7 (6) |  |
| Interpersonal problems |  |  |  |  |  | .042* |
| No | 53.3 (40) | 64.2 (140) | 80.0 (8) | 75.0 (9) | 82.8 (24) |  |
| Yes | 46.7 (35) | 35.8 (78) | 20.0 (2) | 25.0 (3) | 17.2 (5) |  |
| Parenting problems |  |  |  |  |  | .258 |
| No | 73.3 (55) | 78.4 (171) | 70.0 (7) | 75.0 (9) | 93.1 (27) |  |
| Yes | 26.7 (20) | 21.6 (47) | 30.0 (3) | 25.0 (3) | 6.9 (2) |  |
| Living conditions |  |  |  |  |  | .472 |
| No | 66.7 (50) | 72.5 (158) | 70.0 (7) | 83.3 (10) | 82.8 (24) |  |
| Yes | 33.3 (25) | 27.5 (60) | 30.0 (3) | 16.7 (2) | 17.2 (5) |  |
| Turning point stress |  |  |  |  |  | .369 |
| No | 73.3 (55) | 68.8 (150) | 90.0 (9) | 83.3 (10) | 79.3 (23) |  |
| Yes | 26.7 (20) | 31.2 (68) | 10.0 (1) | 16.7 (2) | 20.7 (6) |  |

Note. Values are presented as percentage (n). * p < .05.

Chi-square test p<0.05

Financial stressors were significantly higher in the DU and SU groups at 41.7% (91 individuals) and 50% (6 individuals), respectively, which is approximately twice the rate observed in the control group (20.7%, 6 individuals). Therefore, financial problems were found to be significantly different between groups (p=0.045). Interpersonal problems were higher in the DK and DU groups at 46.7% (35 individuals) and 35.8% (78 individuals), respectively. In the symptomatic groups, interpersonal relationship problems were also higher than in the control group at 20% (2 individuals) and 25% (3 individuals) (p=0.042). No significant differences were found between groups in terms of other life stressors.

### **Table 4.4. Comparison of Brief Symptom Inventory Subcomponents Between Groups**

| Variables | DK % (n) | DU % (n) | SK % (n) | SU % (n) | K % (n) | p |
| --- | --- | --- | --- | --- | --- | --- |
| Total psychopathology |  |  |  |  |  | .<001** |
| No | 42.7 (32) | 29.8 (65) | 0.0 (0) | 0.0 (0) | 100.0 (29) |  |
| Yes | 57.3 (43) | 70.2 (153) | 100.0 (10) | 100.0 (12) | 0.0 (0) |  |
| Anxiety |  |  |  |  |  | .<001** |
| No | 9.3 (7) | 5.5 (12) | 0.0 (0) | 0.0 (0) | 62.1 (18) |  |
| Yes | 90.7 (68) | 94.5 (206) | 100.0 (10) | 100.0 (12) | 37.9 (11) |  |
| Depression |  |  |  |  |  | .<001** |
| No | 41.3 (31) | 25.2 (55) | 0.0 (0) | 0.0 (0) | 89.7 (26) |  |
| Yes | 58.7 (44) | 74.8 (163) | 100.0 (10) | 100.0 (12) | 10.3 (3) |  |
| Negative self |  |  |  |  |  | .<001** |
| No | 12.0 (9) | 11.0 (24) | 0.0 (0) | 0.0 (0) | 51.7 (15) |  |
| Yes | 88.0 (66) | 89.0 (194) | 100.0 (10) | 100.0 (12) | 48.3 (14) |  |
| Somatization |  |  |  |  |  | .<001** |
| No | 54.7 (41) | 41.3 (90) | 30.0 (3) | 8.3 (1) | 86.2 (25) |  |
| Yes | 45.3 (34) | 58.7 (128) | 70.0 (7) | 91.7 (11) | 13.8 (4) |  |
| Hostility |  |  |  |  |  | .<001** |
| No | 45.3 (34) | 35.8 (78) | 0.0 (0) | 8.3 (1) | 93.1 (27) |  |
| Yes | 54.7 (41) | 64.2 (140) | 100.0 (10) | 91.7 (11) | 6.9 (2) |  |
| Hopelessness |  |  |  |  |  | .<001** |
| No | 32.0 (24) | 32.1 (70) | 10.0 (1) | 16.7 (2) | 86.2 (25) |  |
| Yes | 68.0 (51) | 67.9 (148) | 90.0 (9) | 83.3 (10) | 13.8 (4) |  |

Note. Values are presented as percentage (n). ** p < .001.

Chi-square test p<0.05

There were significant differences between groups in all subscales of the Brief Symptom Inventory. Anxiety, depression, and negative self subscales were detected at 100% in the symptomatic group. Somatization and hostility were also higher in the symptomatic group compared to other groups. For all subscales, p=0.000.

Hopelessness was significantly higher in the non-DSM-5 symptomatic group, with rates of 90% (9 individuals) in SK and 83.3% (10 individuals) in SU (p=0.000). Among those diagnosed with DSM-5, hopelessness was also significantly high at 68% (51 individuals) in DK and 67.9% (148 individuals) in DU. Hopelessness was significantly higher in all symptomatic individuals compared to controls (p=0.000).

### **Table 4.5. Comparison of Buss–Perry Aggression Scale Subscales Between Groups**

| Variables | DK % (n) | DU % (n) | SK % (n) | SU % (n) | K % (n) | p |
| --- | --- | --- | --- | --- | --- | --- |
| Physical aggression |  |  |  |  |  | .004* |
| No | 53.3 (40) | 56.9 (124) | 40.0 (4) | 16.7 (2) | 79.3 (23) |  |
| Yes | 46.7 (35) | 43.1 (94) | 60.0 (6) | 83.3 (10) | 20.7 (6) |  |
| Verbal aggression |  |  |  |  |  | .002* |
| No | 50.7 (38) | 49.5 (108) | 40.0 (4) | 8.3 (1) | 75.9 (22) |  |
| Yes | 49.3 (37) | 50.5 (110) | 60.0 (6) | 91.7 (11) | 24.1 (7) |  |
| Irritability |  |  |  |  |  | .<001** |
| No | 48.0 (36) | 42.7 (93) | 20.0 (2) | 16.7 (2) | 82.8 (24) |  |
| Yes | 52.0 (39) | 57.3 (125) | 80.0 (8) | 83.3 (10) | 17.2 (5) |  |
| Hostility |  |  |  |  |  | .<001** |
| No | 48.0 (36) | 46.3 (101) | 20.0 (2) | 8.3 (1) | 82.8 (24) |  |
| Yes | 52.0 (39) | 53.7 (117) | 80.0 (8) | 91.7 (11) | 17.2 (5) |  |

Note. Values are presented as percentage (n). * p < .05, ** p < .001.

Chi-square test p<0.05

Physical aggression (83.3%, 10 individuals), verbal aggression (91.7%, 11 individuals), irritability (83.3%, 10 individuals), and hostility (91.7%, 11 individuals) were highest in the SU group. All groups scored significantly higher than the control group on the Buss–Perry aggression subscales. The p-values were 0.004 for physical aggression, 0.002 for verbal aggression, 0.000 for irritability, and 0.000 for hostility.

### **Table 4.6. Comparison of PTED Self-Rating Scale Items Between Groups**

| Item (Description) | DK % (n) | DU % (n) | SK % (n) | SU % (n) | K % (n) | p |
| --- | --- | --- | --- | --- | --- | --- |
| 01. Exposure to a distressing event | 56.0 (42) / 44.0 (33) | 36.2 (79) / 63.8 (139) | 40.0 (4) / 60.0 (6) | 50.0 (6) / 50.0 (6) | 75.9 (22) / 24.1 (7) | < .001 |
| 02. Impaired well-being | 66.7 (50) / 33.3 (25) | 37.6 (82) / 62.4 (136) | 50.0 (5) / 50.0 (5) | 33.3 (4) / 66.7 (8) | 86.2 (25) / 13.8 (4) | < .001 |
| 03. Sense of injustice | 58.7 (44) / 41.3 (31) | 45.4 (99) / 54.6 (119) | 60.0 (6) / 40.0 (4) | 25.0 (3) / 75.0 (9) | 75.9 (22) / 24.1 (7) | .004 |
| 04. Rumination | 48.0 (36) / 52.0 (39) | 33.9 (74) / 66.1 (144) | 30.0 (3) / 70.0 (7) | 33.3 (4) / 66.7 (8) | 69.0 (20) / 31.0 (9) | .003 |
| 05. Hurt feelings | 46.7 (35) / 53.3 (40) | 36.7 (80) / 63.3 (138) | 40.0 (4) / 60.0 (6) | 33.3 (4) / 66.7 (8) | 65.5 (19) / 34.5 (10) | .038 |
| 06. Revenge thoughts | 76.0 (57) / 24.0 (18) | 73.9 (161) / 26.1 (57) | 60.0 (6) / 40.0 (4) | 33.3 (4) / 66.7 (8) | 93.1 (27) / 6.9 (2) | .002 |
| 07. Self-directed anger | 61.3 (46) / 38.7 (29) | 50.9 (111) / 49.1 (107) | 50.0 (5) / 50.0 (5) | 33.3 (4) / 66.7 (8) | 93.1 (27) / 6.9 (2) | < .001 |
| 08. Giving up | 65.3 (49) / 34.7 (26) | 51.4 (112) / 48.6 (106) | 50.0 (5) / 50.0 (5) | 33.3 (4) / 66.7 (8) | 86.2 (25) / 13.8 (4) | .001 |
| 09. Gloominess/unhappiness | 56.0 (42) / 44.0 (33) | 40.8 (89) / 59.2 (129) | 40.0 (4) / 60.0 (6) | 25.0 (3) / 75.0 (9) | 82.8 (24) / 17.2 (5) | < .001 |
| 10. Physical health impairment | 62.7 (47) / 37.3 (28) | 39.9 (87) / 60.1 (131) | 30.0 (3) / 70.0 (7) | 25.0 (3) / 75.0 (9) | 79.3 (23) / 20.7 (6) | < .001 |
| 11. Avoidance | 69.3 (52) / 30.7 (23) | 53.7 (117) / 46.3 (101) | 60.0 (6) / 40.0 (4) | 25.0 (3) / 75.0 (9) | 93.1 (27) / 6.9 (2) | < .001 |
| 12. Helplessness | 57.3 (43) / 42.7 (32) | 39.4 (86) / 60.6 (132) | 20.0 (2) / 80.0 (8) | 25.0 (3) / 75.0 (9) | 86.2 (25) / 13.8 (4) | < .001 |
| 13. Embitterment | 60.0 (45) / 40.0 (30) | 55.5 (121) / 44.5 (97) | 40.0 (4) / 60.0 (6) | 58.3 (7) / 41.7 (5) | 86.2 (25) / 13.8 (4) | .022 |
| 14. Reduced life drive | 68.0 (51) / 32.0 (24) | 48.2 (105) / 51.8 (113) | 20.0 (2) / 80.0 (8) | 25.0 (3) / 75.0 (9) | 82.8 (24) / 17.2 (5) | < .001 |
| 15. Irritability | 65.3 (49) / 34.7 (26) | 47.2 (103) / 52.8 (115) | 40.0 (4) / 60.0 (6) | 25.0 (3) / 75.0 (9) | 86.2 (25) / 13.8 (4) | < .001 |
| 16. Concentration problems | 62.7 (47) / 37.3 (28) | 48.2 (105) / 51.8 (113) | 40.0 (4) / 60.0 (6) | 50.0 (6) / 50.0 (6) | 82.8 (24) / 17.2 (5) | .003 |
| 17. Disruption of family life | 66.7 (50) / 33.3 (25) | 45.4 (99) / 54.6 (119) | 20.0 (2) / 80.0 (8) | 16.7 (2) / 83.3 (10) | 86.2 (25) / 13.8 (4) | < .001 |
| 18. Reduced social functioning | 66.7 (50) / 33.3 (25) | 49.1 (107) / 50.9 (111) | 70.0 (7) / 30.0 (3) | 25.0 (3) / 75.0 (9) | 93.1 (27) / 6.9 (2) | < .001 |
| 19. Painful memories | 61.3 (46) / 38.7 (29) | 39.0 (85) / 61.0 (133) | 30.0 (3) / 70.0 (7) | 25.0 (3) / 75.0 (9) | 79.3 (23) / 20.7 (6) | < .001 |

Note. Values are presented as percentage (n). DK: DSM-5 diagnosis with duration <6 months; DU: DSM-5 diagnosis ≥6 months; SK: no DSM-5 diagnosis <6 months; SU: no DSM-5 diagnosis ≥6 months; K: control group.

Note. Values are presented as percentage (n). ** p < .001.

Chi-square test p<0.05

All items of the PTED self-rating scale showed significant differences between groups. The third item, “experience of an event causing feelings of injustice,” was most prominent in the SU group at 75% (9 individuals) (p=0.004). The sixth item, “thoughts of revenge,” was also highest in this group at 66.7% (8 individuals) (p=0.002). The highest rates of irritability, sadness, avoidance, helplessness, decreased motivation, deterioration of physical health, and impairment in family and social life were also observed in this group (p<0.05 for all items).

### **Table 4.7. Correlations Between PTED Total Scores and Other Variables**

| Variable | Spearman r | p |
| --- | --- | --- |
| Exposure to distressing event | .743 | < .001 |
| Impaired well-being | .779 | < .001 |
| Sense of injustice | .753 | < .001 |
| Rumination | .800 | < .001 |
| Hurt feelings | .754 | < .001 |
| Revenge thoughts | .617 | < .001 |
| Self-directed anger | .713 | < .001 |
| Giving up | .676 | < .001 |
| Gloominess/unhappiness | .842 | < .001 |
| Physical health impairment | .773 | < .001 |
| Avoidance | .795 | < .001 |
| Helplessness | .831 | < .001 |
| Embitterment | .711 | < .001 |
| Reduced life drive | .780 | < .001 |
| Irritability | .720 | < .001 |
| Concentration problems | .753 | < .001 |
| Family life disruption | .786 | < .001 |
| Reduced social functioning | .752 | < .001 |
| Painful memories | .834 | < .001 |
| Hopelessness | .490 | < .001 |
| Anxiety | .578 | < .001 |
| Depression | .626 | < .001 |
| Negative self | .638 | < .001 |
| Somatization | .496 | < .001 |
| Hostility | .617 | < .001 |
| Physical aggression | .394 | < .001 |
| Verbal aggression | .312 | < .001 |
| Irritability (aggression) | .418 | < .001 |
| Hostile attitude | .465 | < .001 |
| Paranoid ideation | .650 | < .001 |
| Number of stressors | .456 | < .001 |

Note. Spearman correlation coefficients are presented. All correlations are with PTED total score. ** p < .001.

Spearman correlation test p<0.001

Since the PTED total score did not show a normal distribution, Spearman correlation analysis was used. According to the results, the PTED total score was correlated with all variables (p=0.000 for all). The strongest correlations were observed with sulkiness (r=0.842), rumination (r=0.800), and experiencing a painful event (r=0.834). Additionally, embitterment was notably associated with anger, hopelessness, hostility, and depression. A significant correlation was also found between the number of stressors and embitterment.

### **Table 4.8. Gender Differences in Variables Associated with Embitterment in DU and SU Groups**

| Variable | Female % (n) | Male % (n) | p |
| --- | --- | --- | --- |
| Occupation | 45.2 (76) | 47.5 (47) | < .001 |
| Marital stressor | 31.5 (53) | 17.2 (17) | .010 |
| Work-related stressor | 32.7 (55) | 57.6 (57) | < .001 |
| History of abuse | 10.7 (13) | 3.0 (3) | .032 |
| Physical aggression | 37.5 (63) | 60.6 (60) | < .001 |
| Verbal aggression | 46.4 (78) | 64.6 (64) | .005 |
| Irritability | 53.0 (89) | 72.7 (72) | .002 |
| Hostile attitude | 50.0 (84) | 70.7 (70) | .001 |

Note. Values are presented as percentage (n). Chi-square and Fisher’s exact tests were used. DU = DSM-5 diagnosis with duration ≥6 months; SU = no DSM-5 diagnosis with symptoms ≥6 months. p < .05 indicates statistical significance.Chi-square test (Fisher) p<0.05

Among individuals with embitterment disorder, being a housewife (45.2%, 76 individuals, p=0.000) and marital stress (31.5%, 53 individuals, p=0.010) were significant in women. In men, working in the private sector (47.5%, 47 individuals, p=0.000) and job-related stressors (57.6%, 57 individuals, p=0.000) were significantly higher. Abuse was a significant early-life stressor in women at 10.7% (13 individuals) compared to men (p=0.032). All aggression and anger subscale scores were significantly higher in men: physical aggression (p=0.000), verbal aggression (p=0.005), irritability (p=0.002), and hostility (p=0.001). No significant gender differences were found in other variables.

### **Table 4.9. Logistic Regression of Variables with Significant Differences by Gender**

| Variable | Total (p / OR) | Female (p / OR) | Male (p / OR) |
| --- | --- | --- | --- |
| Model accuracy (%) | 94.5 | 96.1 | 90.7 |
| Stressor load | .575 / 1.400 | .920 / 0.928 | .997 / 0.994 |
| Depression | .090 / 3.109 | .038 / 6.182* | .973 / 0.934 |
| Paranoid ideation | .508 / 1.490 | .470 / 1.662 | .910 / 0.819 |
| Anger | .870 / 1.097 | .578 / 0.676 | .593 / 3.773 |
| Rumination | .001 / 7.075* | .003 / 7.922* | .339 / 3.828 |
| Revenge | .001 / 13.965* | .010 / 16.547* | .091 / 28.801 |
| Giving up | .001 / 6.772* | .039 / 4.550* | .152 / 13.706 |
| Gloominess | .032 / 3.254* | .038 / 4.853* | .258 / 9.082 |
| Helplessness | < .001 / 8.432* | .034 / 4.120* | .022 / 40.327* |
| Painful memories | < .001 / 7.908* | .003 / 7.326* | .048 / 47.314* |
| Injustice | .521 / 1.433 | .901 / 1.095 | .153 / 13.477 |
| Hope | .549 / 1.415 | .970 / 0.973 | .219 / 8.364 |
| Marital status | .618 / 0.718 | .657 / 1.416 | .262 / 0.142 |

Note. OR = odds ratio. Logistic regression results are presented as p value / OR. * p < .05.

Logistic regression p<0.05

According to regression analysis, in the overall sample, revenge increased the risk of embitterment disorder approximately 13-fold; helplessness and painful memories increased it approximately 8-fold; rumination and giving up increased it approximately 7-fold; and sulkiness increased it approximately 3-fold.

In women, the presence of depressive symptoms increased the risk approximately 6.1-fold. In men, having painful memories and helplessness increased the risk approximately 40-fold.
